# AMSS-PCR在肺癌突变基因检测中的价值研究

**DOI:** 10.3779/j.issn.1009-3419.2018.11.02

**Published:** 2018-11-20

**Authors:** 柯 金, 绚 谢, 越江 潘, 科喜 王, 柏深 陈, 多光 吴, 卓坚 沈, 铭辉 王, 惠忠 张

**Affiliations:** 510120 广州，中山大学孙逸仙纪念医院胸外科 Department of Thoracic Surgery, Sun Yat-Sen Memorial Hospital of Sun Yat-Sen University, Guangzhou 510120, China

**Keywords:** 一代测序, 突变位点特异扩增法, 扩增阻碍突变系统, 实时定量聚合酶链式反应, 肺癌突变基因检测, Sanger sequencing, Amplification Mutation Specific System, Amplification Refractory Mutation System, Quantitative real-time polymerase chain reaction, Lung cancer gene mutation detection

## Abstract

**背景与目的:**

肺癌驱动基因检测具有重要意义，目前检测方法多样，临床适用性有差异。本研究旨在比较基于扩增阻碍突变系统-聚合酶链反应(Amplification Refractory Mutation System-polymerase chain reaction, ARMS-PCR)技术的试剂盒与一代测序及ARMS-qPCR检测肺癌突变基因的敏感性和特异性，探究突变位点特异扩增法(Amplification Mutation Specific System, AMSS)-PCR技术在肺癌突变基因检测中的应用价值。

**方法:**

对前期已行ARMS-PCR检测的肿瘤标本进行一代测序及试剂盒检测，比较各种方法的检测结果，并对检测结果进行统计学分析。

**结果:**

本研究共收集了309例肺癌标本。试剂盒与一代测序符合率97.41%，ARMS-PCR的符合率97.73%。试剂盒与一代测序、试剂盒与实时定量聚合酶链反应(quantitative real-time polymerase chain reaction, qPCR)、qPCR与一代测序一致性检验的*Kappa*值分别为0.946、0.953、0.913。试剂盒以一代测序为参照的受试者工作特征曲线(receiver operating characteristic curve, ROC)曲线下面积为0.976，以qPCR为参照的ROC曲线下面积为0.975。

**结论:**

AMSS-qPCR技术能够有效检测肺癌突变基因，具有较好的临床应用价值。

## Abstract (en)

**Background and objective:**

The detection of driver oncogenes of lung cancer is of great importance. There are various gene detection techniques nowadays which are different from each other. We carried out this study to investigate the specificity and sensitivity of assay panels based on an Amplification Refractory Mutation System-polymerase chain reaction (ARMS-PCR) technique of Amplification Mutation Specific System (AMSS) in detection of lung cancer gene mutation. To estimate the applicable value of assay panels in clinical settings.

**Methods:**

We collected cancer tissue specimens or fluid specimens from 309 patients. Mutation results were presented for those samples previously detected by ARMS-PCR. In comparison, we carried out AMSS-PCR using (epidermal growth factor receptor, EGFR) assay panel and Six-Alliance assay panel as well as Sanger sequencing. Software SPSS 22.0 (SPSS IBM) was used for statistical analysis.

**Results:**

The rates of consistency between the results by assay panels and Sanger sequencing or ARMS-PCR were 97.41% and 97.73%, respectively. Besides, EGFR assay panel had higher consistency rates with other detection methods than Six-Alliance assay panel. As for consistency test, the *Kappa* values of assay panels with Sanger sequencing, assay panels with ARMS-PCR, and ARMS-PCR with Sanger sequencing were 0.946, 0.953, and 0.913, respectively. The receiver operating characteristic curve (ROC) area under curve (AUC) of assay panels was 0.976 referring to Sanger sequencing, and 0.975 as ARMS-PCR was referred to.

**Conclusion:**

AMSS-PCR can make an optimal cancer gene mutation detection method for clinical settings.

我国肺癌发病率高，国家癌症中心2017年的统计报告显示，全国恶性肿瘤发病第1位的是肺癌，每年新发病例约78.1万，肺癌致死率高，我国每年约59.1万人死于肺癌^[1]^。

近几十年来，随着对肺癌基因学及分子生物学研究的深入，出现了多种类型效果良好的靶向药物^[2]^。这些靶向药物主要针对*EGFR*、*BRAF*、*HER2*等点突变基因，*FGFR*、*MET*扩增以及*ALK*、*ROS1*、*RET*等重排基因变异^[3, 4]^。靶向药物对携带敏感突变基因的患者具有良好的抗肿瘤效果，因此，基因检测在肺癌诊断与治疗中的应用越来越广泛。

基因检测方法多样^[5]^，包括：一代测序(也称为Sanger测序)、二代测序、实时定量聚合酶链反应(quantitative real-time polymerase chain reaction, qPCR)等，qPCR又可分为普通探针、小沟结合(minor groove binder, MGB)探针、扩增阻碍突变系统(Amplification Refractory Mutation System, ARMS)-PCR等多种类型。突变位点特异扩增法(Amplification Mutation Specific System, AMSS)-PCR利用突变碱基及特殊修饰的TaqMan探针，使得突变型有荧光信号，而野生型没有荧光信号。该方法仅用待检样本形成的检测曲线，即可直观判读定性结果，最大限度地减少了误判风险。AMSS-PCR是一种新型的高敏感、高特异性的突变基因检测方法。目前尚无研究报道该检测方法在实际检测中的效用。本研究通过对比Sanger测序、传统ARMS-PCR以及AMSS-PCR在肿瘤突变基因检测中的敏感性、特异性等指标，明确AMSS-PCR在肺癌突变基因检测中的实用价值。

## 1 材料与方法

2017年1月-2018年5月间收集309例肺癌患者组织或体液标本，分别用试剂盒及一代测序进行突变基因检测，并与患者前期所测Ct(cycle threshold)值的ARMS-PCR结果进行比较。利用SPSS 22.0分析数据，对不同检测方法进行一致性*Kappa*检验，绘制分别以一代测序及ARMS-PCR为参照的试剂盒ROC，进一步明确试剂盒检测的敏感性和特异性。

试剂盒：EGFR试剂盒(可检测包括19DEL、L858R、T790M、20ins、C797S等在内的大部分已知EGFR突变)；六联试剂盒(可检测*KRAS*、*BRAF*、*ALK*融合、*ROS1*融合、*MET*、*HER2*基因的主要突变)。

试剂盒采用AMSS-PCR，利用突变碱基及特殊修饰的Taqman探针，使得突变型有荧光信号，而野生型无荧光信号。

两种试剂盒均来自上海桐树生物科技有限公司。

## 2 结果

### 2.1 患者基本情况、标本类型、病理诊断

详见表 1。结果显示，309例患者中男性196例，占63.43%；女性113例，占36.57%。患者年龄范围为23岁-84岁，中位年龄为63岁。所测基因突变306例来源于组织标本，液体标本共3例，其中2例为胸腔积液，1例为静脉血。病理类型信息显示，以非小细胞肺癌(non-small cell lung cancer, NSCLC)为主，占所有病理诊断的94.82%，其中肺鳞状细胞癌占所有病理类型的23.30%，肺腺癌占70.23%。

**1:** 患者基本信息 The baseline information of cases involved

	Number (*n*=309)	Proportion
Gender		
Male	196	63.43%
Female	113	36.57%
Age (range, yr)	23-84	
Types of samples		
Fresh tissue	273	88.35%
Paraffin-embedded tissue	22	7.12%
Aspiration biopy	11	3.56%
Pleural effusion	2	0.65%
Venous blood	1	0.32%
Histological type		
Non-small cell lung cancer	293	94.82%
Squamous cell cancer	72	23.30%
Adenocarcinoma	217	70.23%
Large cell carcinoma	3	0.97%
Adenosquamous carcinoma	1	0.32%
Small cell lung carcinoma	4	1.29%
Carcinoid tumor	1	0.32%
Undefined	11	3.56%

### 2.2 测序结果

根据患者前期ARMS-PCR检测结果，分别用EGFR试剂盒或六联试剂盒验证突变基因，同时进行一代测序，以便分析比较检测结果。试剂盒与一代测序、ARMS-PCR检测结果之间的符合率及详细符合情况分别见表 2、表 3。结果显示：试剂盒与一代测序及ARMS-PCR有极高的符合率。两种试剂盒比较，EGFR试剂盒与其他三种测序方法的符合情况优于六联试剂盒。试剂盒与一代测序符合情况优于ARMS-PCR与一代测序。

**2:** 试剂盒与一代测序以及ARMS-PCR测序结果符合情况 The comparison of detection results between assay panels and Sanger sequencing or ARMS-PCR

		Sanger sequencing				ARMS-PCR		
		Positive	Negative	Consistency rate (%)		Positive	Negative	Consistency rate (%)
EGFR assay panel	Positive	77	2	98.33		79	0	98.89
	Negative	1	100			2	99	
Six-alliance assay panel	Positive	43	4	96.12		45	2	96.12
	Negative	1	81			3	79	
Total	Positive	120	6	97.41		124	2	97.73
	Negative	2	181			5	178	
ARMS-PCR: Amplification Refractory Mutation System-polymerase chain reaction; EGFR: epidermal growth factor receptor.								

**3:** ARMS-PCR与一代测序基因检测结果符合情况 The comparison of detection results between Sanger sequencing and ARMS-PCR

		Sanger sequencing		
		Positive	Negative	Consistency rate (%)
ARMS-PCR	Positive	119	10	95.79
	Negative	3	177	

### 2.3 三组检测方法的一致性

对三组方法的检测结果进行了一致性*Kappa*检验，结果显示：两种试剂盒与一代测序*Kappa*值为0.946，两种试剂盒与ARMS-PCRKappa值为0.953，ARMS-PCR与一代测序Kappa值为0.913。Kappa值≥0.75显示一致性良好，本研究中，三类检验方法一致性均良好。其中，试剂盒与一代测序及ARMS-PCR的一致性均优于ARMS-PCR与一代测序的一致性。

### 2.4 试剂盒检测的敏感性及特异性

分别以一代测序及ARMS-PCR为参照，绘制试剂盒受试者工作特征曲线(receiver operating characteristic curve, ROC)并计算曲线下面积(area under curve, AUC)，结果见图 1。ROC曲线结果显示：无论以作为金标准的一代测序，还是以敏感性较高ARMS-PCR为参照，试剂盒检测均有极高的敏感性与特异性。ROC AUC≥0.9时，说明准确性高。本研究中，试剂盒与一代测序ROC的AUC为0.976，试剂盒与ARMS-PCR ROC的AUC为0.975，准确性良好。

**1:**
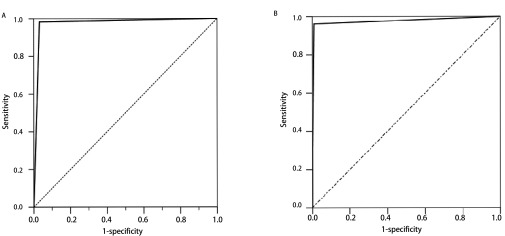
试剂盒检测与一代测序及ARMS-PCR的ROC。A：以一代测序为参照的试剂盒ROC；B：以ARMS-PCR为参照的试剂盒ROC。 ROC for detection results of assay panels referring to Sanger sequencing and ARMS-PCR. A: ROC of assay panels refer to Sanger sequencing (AUC: 0.976); B: ROC of assay panels refer to ARMS-PCR (AUC: 0.975). ROC: receiver operating characteristic curve. AUC: area under curve.

## 3 讨论

一代测序，又称Sanger测序，1977年由Frederick Sanger研发而成，是基于链终止的DNA测序方法。该方法以目标DNA单链为模板，在DNA多聚酶的催化下，合成新链，当荧光标记的双脱氧核苷酸被选择性地合成到新链上时，新链合成终止，从而得到一系列长度不等的DNA片段，再进行电泳，从而完成DNA测序^[6]^。迄今为止，Sanger测序仍然是基因测序的金标准，所有其他测序方法都以Sanger测序做参照。Sanger测序虽然是基因测序的金标准，但也有其局限性，包括：测序效率低，成本高，不能实现短时间内完成大量测序。

qPCR是利用PCR反应检测靶基因的一种检测方法。该检测需要加入靶基因特异性引物，检测过程或掺入一种双链DNA特异性染料，或利用5’-3’核酸外切酶释放Taqman FRET(荧光共振能量转移)探针。20世纪90年代初，qPCR技术迅速发展，利用该项技术可以进行基因分型、基因表达分析、拷贝数变异检测以及病原体检测等多方面检测，因此，在临床与研究机构应用广泛^[7-10]^。qPCR因其高敏感性及特异性，被美国食品药品管理监督总局(Food and Drug Administration, FDA)批准为多种临床基因检测的金标准。李永文等^[11]^研究也证明，与一代测序相比，qPCR更适用于临床检测。

AMSS-PCR基于ARMS、MGB探针以及荧光PCR技术，实现样本DNA和RNA中*EGFR*、*KRAS*、*BRAF*、*HER2*、*MET*、*ALK*、*ROS1*基因突变检测。

ARMS又称等位基因特异性扩增(alleles specific amplification, ASA)。利用PCR引物的3’端末位碱基必须与其模板DNA互补才能有效扩增的原理，设计等位基因特异性PCR扩增引物，在严格的条件下，只有在引物3’碱基与模板配对时才能出现PCR扩增带，从而检测出突变。ARMS-PCR是一种高敏感性、高特异性、低成本的突变检测方法^[12]^。ARMS-PCR还有一个比较显著的优势，就是该项检测设计扩增的DNA片段比普通DNA片段长，不管突变状态如何，突变位点两侧的共同区域都可以被扩增成独立的片段。这些扩增出来的独立片段是相同的，可用做DNA模板质量内参，对PCR反应也有潜在的抑制作用^[13]^。

ARMS-PCR比直接测序有更高的敏感性。一般来说，直接测序需要标本中突变的肿瘤细胞数目占可检测的所有肿瘤细胞数目的20%以上^[14, 15]^，而ARMS-PCR因为使用特异性探针扩增相应的突变序列，标本中突变的肿瘤细胞数目达可检测的所有肿瘤细胞数目的1%即可检测出突变^[16]^。ARMS-PCR的局限性在于，这种检测方法只能用于已知突变的检测，而如果已知突变占所检测位点的绝大部分(如*EGFR*，已知常见突变占所有突变的95%以上)^[17]^，这一局限性就无足轻重了。

qPCR所用探针不同，检测结果也有差异。Taqman探针两端分别带有荧光基团和荧光淬灭基团，在正常状态下，探针上的荧光被淬灭，不发出荧光。开始进行PCR扩增后，探针被PCR酶水解，淬灭基团与荧光基团分离，荧光信号释放出来，然后根据荧光信号强度确认PCR扩增合成的DNA的量。而MGB探针的淬灭基团采用非荧光淬灭基团(non-fluorescent quencher)，本身不产生荧光，可以大大降低本底信号的强度^[18]^。同时，探针上还连接有MGB修饰基团，可以将探针的Tm值提高10 ℃左右。因此，为了获得同样的Tm值，MGB探针可以比普通TaqMan探针设计得更短，既降低了合成成本，也使得探针设计的成功率大幅提高，在模板的DNA碱基组成不理想的情况下，短的探针比长的更容易设计。

本研究中的试剂盒基于ARMS-PCR以及高效一步法逆转录PCR技术，依据引物和探针与突变位点的完全配对，抑制野生型模板的扩增，FAM荧光从而实现*EGFR*、*KRAS*、*BRAF*、*HER2*、*MET*、*ALK*、*ROS1*基因突变的实时检测；另外每一突变检测反应管中还含有检测人类基因保守区域的引物和HEX标记的荧光探针，作为内控用于监控样本DNA和RNA有无正确加入和PCR扩增过程有无异常。本研究采用试剂盒检测，突变型有荧光信号，野生型无荧光信号，与常规ARMS-PCR不同，无须通过Ct值大小确定检测结果，只要扩增曲线有抬高，即认定为检测结果阳性，降低了误判风险。

研究结果显示，试剂盒与一代测序及测Ct值的ARMS-PCR的符合率高于ARMS-PCR与一代测序的符合率。两种试剂盒比较，EGFR试剂盒与其他三种测序方法的符合情况优于六联试剂盒，考虑与六联试剂盒所检测的基因种类多，只能覆盖各基因的主要突变类型有关。*Kappa*一致性检验及ROC结果显示，试剂盒检测的敏感性及特异性与一代测序及ARMS-PCR测序相当。

基于AMSS-PCR的试剂盒检测方便、快捷，检测结果可靠，与一代测序特异性相当，敏感性高于一代测序；与通过Ct值判定检测结果的ARMS-PCR的敏感性相当，特异性优于高敏感性检测，是适合于临床推广的基因检测方法。

## References

[b1] 1The 2017 updated data on cancer in Chinese population. Zhongguo Zhong Liu Lin Chuang Yu Kang Fu, 2017, 24(6): 760.http://www.cqvip.com/QK/98290X/201705/672369966.html2017年中国最新癌症数据.中国肿瘤临床与康复, 2017, 24(6): 760.

[2] Hirsch FR, Scagliotti GV, Mulshine JL (2017). Lung cancer: current therapies and new targeted treatments. Lancet.

[3] Ortiz-Cuaran S, Scheffler M, Plenker D (2016). Heterogeneous mechanisms of primary and acquired resistance to third-generation EGFR inhibitors. Clin Cancer Res.

[4] Li T, Kung HJ, Mack PC (2013). Genotyping and genomic profiling of non-small-cell lung cancer: implications for current and future therapies. J Clin Oncol.

[5] Köhn L, Johansson M, Grankvist K (2017). Liquid biopsies in lung cancer-time to implement research technologies in routine care?. Ann Transl Med.

[6] Sanger F, Nicklen S, Coulson AR (1997). DNA sequencing with chain-terminating inhibitors. Proc Natl Acad Sci U S A.

[7] Morin PA, McCarthy M (2007). Highly accurate SNP genotyping from historical and low-quality samples. Mol Ecol Notes.

[8] VanGuilder HD, Vrana KE, Freeman WM (2008). Twenty-five years of quantitative PCR for gene expression analysis. BioTechniques.

[9] Weaver S, Dube S, Mir A (2010). Taking qPCR to a higher level: Analysis of CNV reveals the power of high throughput qPCR to enhance quantitative resolution. Methods.

[10] Sedlak RH, Cook L, Cheng A (2014). Clinical utility of droplet digital PCR for human cytomegalovirus. J Clin Microbiol.

[11] Li YW, Liu HY, Li Y (2009). The comparison of real-time PCR with sequencing technique in *EGFR* mutation detection in NSCLC. Zhongguo Fei Ai Za Zhi.

[12] Chen Q, Lu P, Jones AV (2007). Amplification refractory mutation system, a highly sensitive and simple polymerase chain reaction assay, for the detection of *JAK2* V617F mutation in chronic myeloproliferative disorders. J Mol Diagn.

[13] Huang TG, Zhuge J, Zhang WY (2013). Sensitive detection of BRAF V600E mutation by Amplification Refractory Mutation System (ARMS)-PCR. Biomarker Res.

[14] Li J, Wang L, Mamon H (2008). Replacing PCR with COLD-PCR enriches variant DNA sequences and redefines the sensitivity of genetic testing. Nat Med.

[15] Ogino S, Kawasaki T, Brahmandam M (2005). Sensitive sequencing method for *KRAS* mutation detection by pyrosequencing. J Mol Diagn.

[16] Zhou S, Zhou M, Peng H (2014). Comparison of ARMS and direct sequencing for detection of *EGFR* mutation and prediction of EGFR-TKI efficacy between surgery and biopsy tumor tissues in NSCLC patients. Med Oncol.

[17] Castellanos E, Feld E, Horn L (2016). Driven by mutations: the predictive value of mutation subtype in *EGFR*-mutated non-small cell lung cancer. J Thorac Oncol.

[18] Kutyavin IV, Afonina IA, Mills A (2000). 3'-minor groove binder-DNA probes increase sequence specificity at PCR extension temperatures. Nucleic Acids Res.

